# Edaphostat: interactive ecological analysis of soil organism occurrences and preferences from the Edaphobase data warehouse

**DOI:** 10.1093/database/bax080

**Published:** 2017-10-24

**Authors:** Jonas Hausen, Björn Scholz-Starke, Ulrich Burkhardt, Stephan Lesch, Sebastian Rick, David Russell, Martina Roß-Nickoll, Richard Ottermanns

**Affiliations:** 1 Institute for Environmental Research, RWTH Aachen University, Worringerweg 1, 52074 Aachen, Germany; 2 Senckenberg Museum of Natural History Görlitz, P.O. Box 300154, 02806 Görlitz, Germany

## Abstract

The Edaphostat web application allows interactive and dynamic analyses of soil organism data stored in the Edaphobase data warehouse. It is part of the Edaphobase web application and can be accessed by any modern browser. The tool combines data from different sources (publications, field studies and museum collections) and allows species preferences along various environmental gradients (i.e. C/N ratio and pH) and classification systems (habitat type and soil type) to be analyzed.

**Database URL:** Edaphostat is part of the Edaphobase Web Application available at https://portal.edaphobase.org

## Introduction

The analysis of data from ecological databases is a complex process, which requires several consecutive steps and careful adjustments of statistical methods according to the nature of the data and enabling data enrichment and reuse from multiple sources (1). The structure of heterogeneous data within a database is often not appropriate for methods used in classical biological analysis, and because these analyses are made for smaller, closed homogeneous data sets, they often fail to use the full potential of the database. Additionally, for the user of a database, it is often difficult to correctly interpret queried data because of the complexity and amount of data.

Edaphobase is a data warehouse that combines data on soil organisms occurrences with site-specific parameters allowing for the exploration of species distribution and ecological requirements (2). At present, Edaphobase contains data on Nematoda, Oligochaeta (Enchytraeidae and Lumbricidae), Myriapoda (Chilopoda and Diplopoda), Microarthropods (Collembola, Gamasina and Oribatida), Isopoda, and others (Table 1). The project went online in 2012 with a focus of data record distribution on Germany and neighboring countries; however, meanwhile data from all over the world are incorporated. The data warehouse includes compiled data from heterogeneous sources such as literature and museum collections as well as original data from scientific investigations provided by soil fauna researchers and institutions. One of the key objectives of the development of the Edaphobase data warehouse is ‘to provide research tools for access and analysis of data for detailed ecological and biogeographical investigations’ as well as to develop ‘tools for predictions of future changes in soil biological communities …’ (2).

**Table 1.** Taxonomic and geographic data coverage within Edaphobase (acc. 2017-08-29). Taxon coverage depicts the five taxa with the highest percentage of data records in Edaphobase. Geographic coverage shows the five countries, where the most records in Edaphobase are geographically located

Taxon coverage		Geographic coverage	
	% of data records		% of data records
Microarthropods (springtails, moss mites, gamasina mites)	43.9	Germany	76.5
Myriapoda (centipedes and millipedes)	32.6	Austria	2.2
Nematoda (threadworms)	11	Poland	2.0
Oligochaeta (earthworms and microannelids)	8.4	UK	1.8
Isopoda (woodlice)	1.6	Italy	1.6
Other taxa	2.5	Other countries	15.9

Edaphobase allows, after user registration, access to all contained data records except sensitive data partly embargoed according to data providers’ specifications. There are currently over 530000 data records freely available (acc. 2017-10-03). All accessible data is also downloadable.

Vertically integrated databases like Edaphobase combine data from a common topic collected by different sources or studies and include different types of data (taxonomical data, spatial data, environmental parameters, etc.). Although such databases facilitate meta-analyses and data reusability, they are typically more complex than project-specific databases (3). Therefore, extensive preprocessing including transformation, cleaning, and filtering of the data is necessary (4), typically done by hand in classical data analysis. Additionally, certain background knowledge about included studies, taxa groups, etc., is needed for assessment of comparability of data from multiple studies.

**Figure 1.**
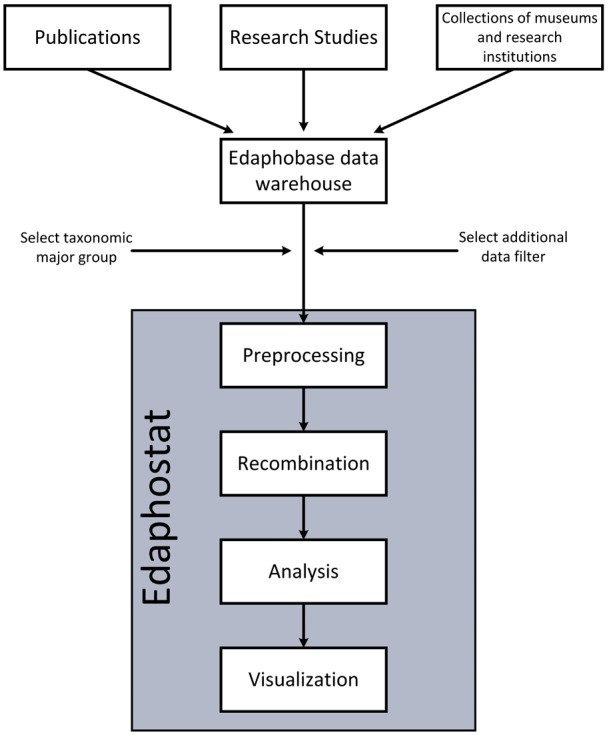
Workflow of Edaphostat. Data from different sources, which are stored in the Edaphobase data warehouse can be selected and filtered. Edaphostat queries these data, prepares and analyzes it automatically.

Here, we present Edaphostat, an interface for an automated analysis and visualization of Edaphobase data for characterizing species environmental preferences. Edaphostat provides a workflow suited for data queried from the Edaphobase warehouse, allowing a maximum of flexibility within the analysis while keeping the necessity of data preprocessing and recombination for the user to a minimum (Figure 1). The workflow is set up and adjusted for the main taxa covered in Edaphobase (Table 1) by domain experts in biodiversity research. This integration of expert knowledge into Edaphostat allows the results to be more intuitive and useful in scientific research. Edaphostat is created for analysis of species preferences along environmental parameter gradients. It is able to process large numbers of heterogeneous records from mixed origins ‘on the fly’ and can thereby exploit the full potential of the database.

## Tool description

Edaphostat is fully written in R (5). It uses the R packages shiny (6), shinydashboard (7) and plotly (8, 9) to create an interactive GUI as well as output graphs. Besides that, the tool combines various functions from other packages for its analysis, but because it runs on a server, there is no need for the user to download R packages or any new code. Edaphostat is publicly available as part of the Edaphobase web application (https://portal.edaphobase.org; 2) and can be accessed by any modern Web browser (select ‘Edaphostat’ in the ‘Tools’ context menu and start the tool through the setup menu). Online manuals for the Edaphobase web application (10) and Edaphostat (11) are available. Because Edaphostat uses the Edaphobase data warehouse and web application, data can be selected and filtered prior to analysis using the Edaphobase Web application (refer to Burkhardt *et al.* and the online manual for usage of the Edaphobase Web application).

The selection dialogue offers the user the choice between presence–absence data and methodological-dependent measures of quantity (e.g. depending on sampling methods: abundance, dominance, etc.). These data are then used in two types of analysis: data for bar charts are classified either by a numeric environmental parameter (e.g. C/N ratio) or by a non-numeric categorical grouping (e.g. habitat classes). The bar charts for the analysis of environmental parameters measured on both numerical and categorical scales allow indication of the response of each taxon to a single environmental variable.

**Figure 2.**
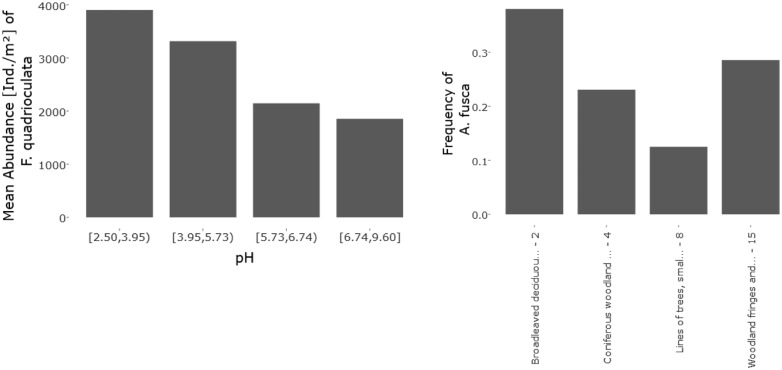
Numeric bar chart of the mean abundance [Ind/m^2^] of Folsomia quadrioculata (Tullberg, 1871) (Hexapoda: Collembola) alongside a pH gradient (left) and non-numeric bar chart of the frequency of Allacma fusca at different EUNIS habitat types (right).

For each class, the frequency and the mean of the chosen quantity are determined and depicted as bar height. The frequency is calculated as the number of sites where the species was found divided by the total number of all sampled sites. Classes (depicted on the x-axis) either were given by the non-numeric classification system (Figure 2, right) or build by grouping the values of the numeric environmental parameter (Figure 2, left). Here, the users can either determine the classes themselves or choose between different classification methods, which are selected using domain expert knowledge.

**Figure 3.**
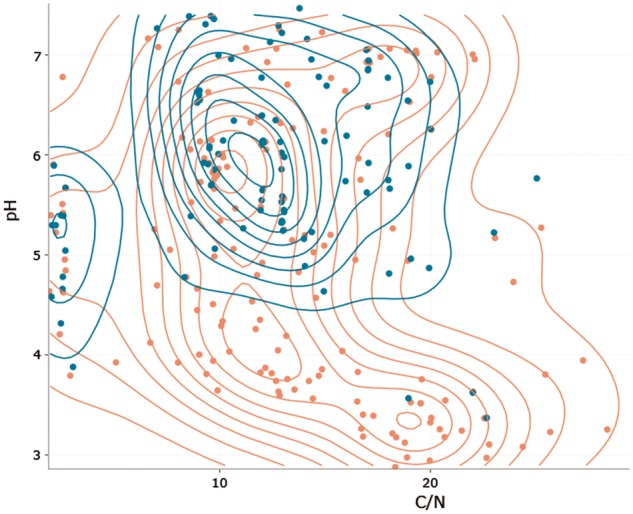
Niche space diagram of Folsomia quadrioculata (Tullberg, 1871) (red) and Isotoma viridis (Bourlet, 1839) (blue; both Hexapoda: Collembola) in response to C/N ratio and pH. Each point represents one site, where one of the species was found. Density lines are added for both species to indicate centroids of occurrence.

A niche-space diagram (Figure 3) compares species occurrences alongside two numeric environmental parameters to uncover typical specializations. The user has the option to add density lines as a visual help to identify a species centroid, which corresponds with its niche. It is also possible to include a second species for means of comparison as well as absence values in the niche space diagram. All graphs are interactive (allowing hover, zoom, etc.) and can be saved and printed as Portable Network Graphics (png). Please refer to the Edaphostat manual (11) for information on how to work with the graphs.

## Scope of application

**Figure 4.**
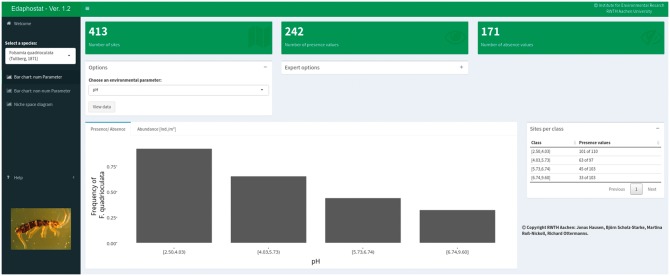
Numeric bar chart module showing the results for Folsomia quadrioculata (Tullberg, 1871) (Hexapoda: Collembola) and pH.

Edaphostat is especially useful for determining species autecological requirements concerning soil or site properties. For this purpose, the user chooses a species of interest as well as the type of analysis from the sidebar on the left. In the next step, the user has to select an environmental parameter (either numeric or non-numeric). If, e.g. users wish to gain insight into the distribution of a species along a C/N gradient, they can do so by choosing bar charts for numerical parameters (Figure 4).

An overview and quick quality assessment of the selected data subset from Edaphobase is provided by three boxes at the top. It shows the number of sites where the chosen parameter (Figure 4: pH) was measured and the taxonomic group was sampled, and within these sites where the selected species was recorded and where it was absent. A bar chart and corresponding table with sites per class allow the user to obtain a detailed view of species occurrences within the various pH classes. It is possible to use the frequency or mean abundance (or any other chosen quantity) as bar height. This is especially useful for defining the type or shape of response for specific species, one of the key problems of ecological niche modeling and niche theory (12–14).

A further analysis could be to determine habitat preferences of the selected species, which can be performed using bar charts for categorical data. A typical result could be a high mean abundance in forests compared to arable or grassland sites. Finally, the user can compare preferences of the selected species with a second species along a C/N ratio and pH gradient using a niche space diagram. This can be used, i.e. to detect ecological differences between closely related species. All analyses can be used as a basis for biological soil quality assessment and derivation of reference values, as done by, e.g. Jänsch *et al.* (15), or Römbke *et al.* (16).

Edaphostat was programmed to automatically analyze data from the Edaphobase data warehouse. Edaphobase combines data from various sources including publications, unpublished data from field studies as well as collections from German research institutions. In contrast to most other biological online analysis tools, Edaphostat performs preprocessing steps to ensure that such, heterogeneous data can be jointly analyzed. During import, data sets are structured using predefined lists and value classes, thus making them suitable for meta-analyses across multiple data sets for further analyses (data-reuse). Thereby, autecological analysis with data from various sources is possible without the need for the user to perform complicated data merging and transformation steps, which oftentimes need profound knowledge about the data and corresponding studies.

One of the recently developed tools for biodiversity research is VAT (17), which analyses data from the German Federation for Biological Data (GFBio). The GFBio data warehouse includes data from all biological and environmental research and also includes Edaphobase data. However, VAT is focused on spatio-temporal analysis (18), whereas Edaphostat is programmed to use the Edaphobase data for autecological analysis. VAT allows the combination of different GFBio datasets (e.g. species distribution and environmental parameters) as layers of a map. While it gives the user the option to create histograms and execute R code, each layer/dataset can only be analyzed separately. Edaphostat allows combined analysis of species occurrences and environmental parameters as variables in the same visualization. This opens the opportunity to use all Edaphobase data in automated autecological analysis. To our knowledge at the moment, no other tool exists to combine data sets for the analysis and create, e.g. visualizations of species occurrences alongside an environmental parameter.

The results of Edaphostat are highly dependent on the quality and amount of data that is available through the Edaphobase data warehouse. Even though the tool is programmed for recombination and analysis of highly heterogeneous data sources, results get more reliable and applicable with larger amounts of high quality data. Further additions for Edaphobase will, therefore, include the option to select certain data sets using a Digital Object Identifier (DOI). As soon as this is available, Edaphostat benefits from the ability to analyze well-defined, high-quality data. Further development of the Edaphobase data warehouse aims towards higher data quality by implementing metadata standards protocols into the data import process. If these standards are applied and Edaphostat has been further developed to using them to their full potential, Edaphostat can be used to analyze other databases, which follow the same protocols.

## Example of application

*Folsomia candida* (Willem, 1902) has been used in soil ecotoxicology for a number of years as a ‘standard’ test organism for estimating effects of environmental pollutants (19). Recently, International Standards Organization (ISO) has published the protocol for using *F. candida* as a test species for ‘evaluating the habitat function and determining effects of soil contaminants and substances’ (20). Additionally, *F. candida* is increasingly used in ecological and ecotoxicological modeling as a model organism (21).

Because of the relevance in soil assessment, soil property optima (e.g. C/N ratio) of *F. candida* are important to know in order to distinguish between effects of soil properties like changing pH and the effect of environmental pollutants. Additionally, these parameters are relevant in choosing appropriate growth and test media for laboratory tests.

**Figure 5.**
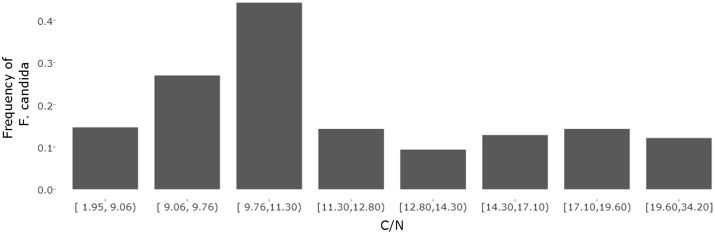
Numerical bar chart of the frequency of Folsomia candida (Willem, 1902) alongside a C/N gradient with eight classes (default = 4)

Edaphostat can be used to determine the species distribution alongside environmental gradients and can thereby help in determining the species optima. Figure 5 shows the frequency of *F. candida* alongside a C/N gradient. The bar chart was generated using the numeric bar chart module from Edaphostat (Figure 4). All sites in Germany where *F. candida* was sampled were used in the analysis (acc. 2017-04-10). The bar chart suggests that *F. candida* has its C/N optimum between 9.5 and 11.5. In soils with lower or higher C/N ratio, it was abundant with a much lower frequency indicating worse conditions for reproduction. For the usage of *F. candida* as a test organism, this means that C/N ratio has to be kept constant or should at least be taken into account when comparing the reproduction of this species in different soil samples. Here, using Edaphostat helps to estimate the effect of a changing C/N ratio on the frequency of *F. candida* and is able to predict an optimum.
